# Pediatric COVID-19 Cases in Counties With and Without School Mask Requirements — United States, July 1–September 4, 2021

**DOI:** 10.15585/mmwr.mm7039e3

**Published:** 2021-10-01

**Authors:** Samantha E. Budzyn, Mark J. Panaggio, Sharyn E. Parks, Marc Papazian, Jake Magid, M Eng, Lisa C. Barrios

**Affiliations:** ^1^CDC COVID-19 Response Team; ^2^Booz Allen Hamilton Corporation, McLean, Virginia; ^3^Johns Hopkins University Applied Physics Laboratory, Laurel, Maryland; ^4^Palantir Technologies, Denver, Colorado.

Consistent and correct mask use is a critical strategy for preventing the transmission of SARS-CoV-2, the virus that causes COVID-19 (*1*). CDC recommends that schools require universal indoor mask use for students, staff members, and others in kindergarten through grade 12 (K–12) school settings (*2*). As U.S. schools opened for the 2021–22 school year in the midst of increasing community spread of COVID-19, some states, counties, and school districts implemented mask requirements in schools. To assess the impact of masking in schools on COVID-19 incidence among K–12 students across the United States, CDC assessed differences between county-level pediatric COVID-19 case rates in schools with and without school mask requirements.

Using data from July 1–September 4, 2021, counties that met the following criteria were included in the analysis: 1) a valid school start date, and MCH Strategic Data* included a known school mask requirement for at least one district; 2) in districts with known school mask requirements, a uniform mask requirement for all students or no students; and 3) at least 3 weeks with 7 full days of case data since the start of the 2021–22 school year. For counties with multiple school districts, the median school start date was used. Counties with conflicting school mask requirements were excluded from this analysis; only those counties with the same known mask requirements for all schools were included. Among the 3,142 U.S. counties included in the initial sample, 16.5% (520) were included in the final analysis after applying the selection criteria. County-specific pediatric COVID-19 rates (number of cases per 100,000 population aged <18 years) from CDC’s COVID Data Tracker^†^ were tabulated and aggregated by school start week. To account for the variation in the weeks each county started school, weeks were numbered from −3 to 2; the school start date was the beginning of week 0. Aggregated pediatric COVID-19 case counts and rates were calculated; average weekly changes were compared for counties with and without school mask requirements using a one-sided t-test. To further assess the association between pediatric COVID-19 cases and school mask requirements, a multiple linear regression was constructed that adjusted for age, race and ethnicity,^§^ pediatric COVID-19 vaccination rate, COVID-19 community transmission, population density, social vulnerability index score,^¶^ COVID-19 community vulnerability index score,** percentage uninsured, and percentage living in poverty. Statistical analyses were completed using SciPY (version 1.2.1) and Statsmodels (version 0.11) analysis modules for Python (version 3.7.6; Python Software Foundation). Statistical significance was defined as p<0.05 for all analyses. This activity was reviewed by CDC and was conducted consistent with applicable federal law and CDC policy.^††^

Counties without school mask requirements experienced larger increases in pediatric COVID-19 case rates after the start of school compared with counties that had school mask requirements (p<0.001) (Figure). The average change from week −1 (1–7 days before the start of school) to week 1 (7–13 days after the start of school) for counties with school mask requirements (16.32 cases per 100,000 children and adolescents aged <18 years per day) was 18.53 cases per 100,000 per day lower than the average change for counties without school mask requirements (34.85 per 100,000 per day) (p<0.001). Comparisons between pediatric COVID-19 case rates during the weeks before (weeks −3, −2, and −1) and after (weeks 0, 1, and 2) the start of school indicate that counties without school mask requirements experienced larger increases than those with school mask requirements (p<0.05). After controlling for covariates, school mask requirements remained associated with lower daily case rates of pediatric COVID-19 (β = −1.31; 95% confidence interval = −1.51 to −1.11) (p<0.001).

**FIGURE:**
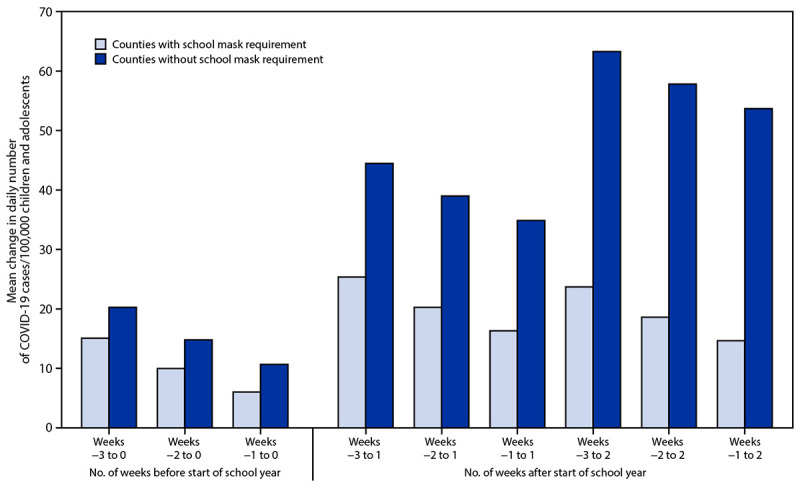
Mean county-level change in daily number of COVID-19 cases per 100,000 children and adolescents aged <18 years in counties (N = 520) with and without school mask requirements* before and after the start of the 2021–22 school year — United States, July 1–September 4, 2021 * Among 520 counties, 198 (38%) had a school mask requirement and 322 (62%) did not have a school mask requirement.

The findings in this report are subject to at least four limitations. First, this was an ecologic study, and causation cannot be inferred. Second, pediatric COVID-19 case counts and rates included all cases in children and adolescents aged <18 years; later analyses will focus on cases in school-age children and adolescents. Third, county-level teacher vaccination rate and school testing data were not controlled for in the analyses; later analyses will control for these covariates. Finally, because of the small sample size of counties selected for the analysis, the findings might not be generalizable.

The results of this analysis indicate that increases in pediatric COVID-19 case rates during the start of the 2021–22 school year were smaller in U.S. counties with school mask requirements than in those without school mask requirements. School mask requirements, in combination with other prevention strategies, including COVID-19 vaccination, are critical to reduce the spread of COVID-19 in schools (*2*).

## Footnotes

* MCH Strategic Data are obtained from a weekly phone survey of public, private, and independent U.S. school districts. MCH surveys schools from the school districts with student enrollment >10,000 (largest districts), 5,000–10,000 (large districts), 1,000–4,999 (medium districts), and <1,000 (small districts). https://www.mchdata.com/covid19/schoolclosings

†
https://covid.cdc.gov/covid-data-tracker/#demographicsovertime

§ Age, race, ethnicity, population density, percent uninsured, and percentage in poverty data are from 2019 U.S. Census estimates (https://www.census.gov/data/datasets/time-series/demo/popest/2010s-counties-total.html) and the 2018 American Community Survey (https://www.census.gov/acs/www/data/data-tables-and-tools/geographic-comparison-tables/).

¶ The social vulnerability index score is a percentile ranking in which a value of 1 indicates the highest risk level. https://svi.cdc.gov/

** The COVID-19 community vulnerability index score is a percentile ranking in which a value of 1 indicates the highest risk level. https://precisionforcovid.org/ccvi

†† 45 C.F.R. part 46.102(l)(2), 21 C.F.R. part 56; 42 U.S.C. Sect. 241(d); 5 U.S.C.0 Sect.552a; 44 U.S.C. Sect. 3501 et seq.
